# Interleukin-2 enhancer binding factor 2 interacts with the nsp9 or nsp2 of porcine reproductive and respiratory syndrome virus and exerts negatively regulatory effect on the viral replication

**DOI:** 10.1186/s12985-017-0794-5

**Published:** 2017-07-11

**Authors:** Xuexia Wen, Ting Bian, Zhibang Zhang, Lei Zhou, Xinna Ge, Jun Han, Xin Guo, Hanchun Yang, Kangzhen Yu

**Affiliations:** 1 Key Laboratory of Animal Epidemiology of the Ministry of Agriculture, College of Veterinary Medicine and State Key Laboratory of Agrobiotechnology, China Agricultural University, No. 2 Yuanmingyuan West Road, Haidian District, Beijing, 100193 People’s Republic of China; 2 The Chinese Ministry of Agriculture, No.11 Nongzhanguan Nanli, Chaoyang District, Beijing, 100125 People’s Republic of China

**Keywords:** Porcine reproductive and respiratory syndrome virus (PRRSV), Nonstructural protein 9 (nsp9), Nonstructural protein 2 (nsp2), Interleukin-2 enhancer binding factor 2 (ILF2), Interaction, Replication

## Abstract

**Background:**

Porcine reproductive and respiratory syndrome virus (PRRSV) causes reproductive failures in sows and respiratory diseases in growing pigs, resulting in huge economic loss for the pig production worldwide. The nonstructural protein 9 (nsp9) and nonstructural protein 2 (nsp2) of PRRSV are known to play important roles in viral replication. Cellular interleukin-2 enhancer binding factor 2 (ILF2) participates in many cellular pathways and involves in life cycle of some viruses. In the present study, we analyzed the interaction of cellular ILF2 with the nsp9 and nsp2 of PRRSV in vitro and explored the effect of ILF2 on viral replication.

**Methods:**

The interaction of ILF2 with the nsp9 or nsp2 of PRRSV was analyzed in 293FT cells and MARC-145 cells by co-immunoprecipitation (Co-IP) and the co-localization of ILF2 with the nsp9 or nsp2 of PRRSV in MARC-145 cell and pulmonary alveolar macrophages (PAMs) was examined by confocal immunofluorescence assay. The effect of ILF2 knockdown and over-expression on PRRSV replication was explored in MARC-145 cells by small interfering RNA (siRNA) and lentivirus transduction, respectively.

**Results:**

The interaction of ILF2 with nsp9 or nsp2 was first demonstrated in 293FT cells co-transfected with ILF2-expressing plasmid and nsp9-expressing plasmid or nsp2-expressing plasmid. The interaction of endogenous ILF2 with the nsp9 or nsp2 of PRRSV was further confirmed in MARC-145 cells transduced with GFP-nsp9-expressing lentiviruses or infected with PRRSV JXwn06. The RdRp domain of nsp9 was shown to be responsible for its interaction with ILF2, while three truncated nsp2 were shown to interact with ILF2. Moreover, we observed that ILF2 partly translocated from the nucleus to the cytoplasm and co-localized with nsp9 and nsp2 in PRRSV-infected MARC-145 cells and PAMs. Finally, our analysis indicated that knockdown of ILF2 favored the replication of PRRSV, while over-expression of ILF2 impaired the viral replication in MARC-145 cells.

**Conclusion:**

Our findings are the first to confirm that the porcine ILF2 interacts with the nsp9 and nsp2 of PRRSV in vitro, and exerts negatively regulatory effect on the replication of PRRSV. Our present study provides more evidence for understanding the roles of the interactions between cellular proteins and viral proteins in the replication of PRRSV.

**Electronic supplementary material:**

The online version of this article (doi:10.1186/s12985-017-0794-5) contains supplementary material, which is available to authorized users.

## Background

Porcine reproductive and respiratory syndrome virus (PRRSV) causes reproductive failures in sows and respiratory diseases in growing pigs, resulting in huge economic loss for the pig production worldwide [1–3]. The PRRSV is an enveloped, single-stranded positive sense RNA virus, which is classified into the order *Nidovirales*, family *Arteriviridae*, genus *Arterivirus* [4, 5]. The genome of this virus is approximately 15 kb in size and contains at least 12 overlapping open reading frames (ORFs), including ORF1a, ORF1b, ORF2a, ORF2b, ORFs 3 to 7 and ORF5a [6–12].

The ORF1a and ORF1b encode 4 known replicase polyproteins (pp1a, pp1a-nsp2N, pp1a-nsp2TF, and pp1ab) [13], and the replicase polyproteins are postranslationally processed into at least 16 distinct nonstructural proteins (nsp), mainly including nsp1α, nsp1β, nsps2 to 6, nsp7α, nsp7β, and nsps8 to 12 [7, 14–17]. The remaining ORFs encode the structural proteins of PRRSV [9, 12, 18–22]. Interestingly, the nsp2 is newly recognized to be an integral membrane protein of PRRSV as a structural protein [23, 24]. PRRSV strains worldwide can be classified into two genotypes, the European type (type 1) and the North American type (type 2) [25, 26]. The viruses of type 1 can be further divided into different subtypes, while type 2 can be differentiated into distinct genetic lineages due to the broad genetic variation and diversity of isolates [27].

The PRRSV nsps have been considered to be involved in viral replication and genome transcription [16], and in the modulation of host innate immune responses [28–32]. Of the PRRSV nsps, the nsp9, the viral RNA-dependent RNA polymerase (RdRp), is considered to be a key enzyme for RNA-templated RNA synthesis [16]. It has been shown to play important roles in the replication efficiency, pathogenicity and virulence of the Chinese highly pathogenic PRRSV (HP-PRRSV) [33], and viral replication regulation via the interaction with cellular host proteins [34–36]. Thus, it is essential to further explore the host cellular proteins interacting with the PRRSV nsp9 and analyze the biological significance of their interaction on the virus life cycle. Therefore, the immunoprecipitation (IP) combined with LC-MS/MS assay was performed to explore host cellular proteins interacting with nsp9. The nsp2, the largest nonstructural protein of PRRSV, is considered as a multifunctional protein in viral replication and pathogenesis [37, 38]. The nsp2, combined with the nsp3, comprises viral RNA synthesis site by inducing double membrane vesicle (DMV) formation [39]. Also, it potentially interacts with nsp1α, nsp1β, nsp3, nsp4, nsp7, nsp9, and nsp10 [40], which are all considered as important components of viral replication and transcription complex (RTC) for viral RNA synthesis [16, 41]. Given the important role of nsp2 in PRRSV replication, it is conductive to better understanding the viral replication process to identify interacting partner of nsp2.

The cellular protein_interleukin-2 enhancer binding factor 2 (ILF2), also named as nuclear factor 45 (NF45) in human and mouse, is initially defined as crucial transcription factors required for interleukin-2 expression during T-cell activation in mammals together with interleukin-2 enhancer binding factor 3 (ILF3), also known as nuclear factor 90 (NF90) in human and mouse [42]. Usually, ILF2 forms a heterodimeric complex with ILF3, which is shown to be implicated in DNA repair [43], gene transcription [44], microRNA processing [45, 46], and mRNA translation [47, 48]. Moreover, ILF2, ILF3 or the ILF2/ILF3 heterodimer has also been involved in various virus life cycle including hepatitis C virus (HCV) [49], infectious bursal disease virus (IBDV) [50], poliovirus [51], influenza virus [52, 53], dengue virus [54], human immunodeficiency virus type 1 (HIV-1) [55], human T-cell leukemia virus [56], as well as in the host defense mechanism protecting from viral infections [57]. Our previous study has screened that ILF3 interacts with the nsp2 of PRRSV JXwn06 [58]. Given that ILF2 usually forms a heterodimeric complex with ILF3 [42], it led us to propose ILF2 may also interact with nsp2. Thus, the aim of present study is to analyze the interaction of cellular ILF2 with the nsp9 and nsp2 of PRRSV in vitro and to explore the effect of their interactions on viral replication.

## Methods

### Cells, virus and antibodies

Both MARC-145 cells and human embryonic kidney 293FT cells were cultured in GIBCO Dulbecco’s modified Eagle medium DMEM (Fisher Scientific, Waltham, MA, USA) supplemented with 10% fetal bovine serum (FBS) in a humidified 5% CO_2_ atmosphere at 37 °C. Porcine pulmonary alveolar macrophages (PAMs) were prepared as previously described [59]. PAMs and 3D4/21 cells (ATCC CRL-2843), a PAM-derived cell line, were both maintained in RPIM-1640 (Fisher Scientific) containing 10% FBS. The stock of PRRSV strain JXwn06 with a titer of 10^7^ TCID_50_/ml was used in this study [60]. Mouse anti-HA monoclonal antibody (mAb) (H3663), mouse anti-β-actin mAb (A5441), rabbit anti-Myc polyclonal antibody (C3956), rabbit anti-GFP polyclonal antibody (G1544) were all purchased from Sigma-Aldrich (St. Louis, MO, USA). Mouse anti-ILF2 mAb was purchased from Santa Cruz Biotechnology (Santa Cruz, CA, USA). Rabbit anti-ILF2 polyclonal antibody and rabbit anti-ILF3 polyclonal antibody were purchased from Proteintech Group (Chicago, IL, USA). The mAbs specific for the nsp9, nsp2 and N protein of PRRSV were prepared in our laboratory.

### Plasmid construction

All plasmids were constructed by standard recombined DNA techniques. Briefly, the nsp9 gene of PRRSV JXwn06 was amplified by PCR from the plasmid pWSK-JXwn06 [60] and then cloned into the vector pWPXL (Addgene, Cambridge, MA, USA) to generate the recombinant plasmid pWPXL-nsp9. The pCMV-HA-nsp9, pCMV-Myc-nsp9 and the truncated nsp9-expressing plasmids were prepared in our laboratory [36]. The gene coding for ILF2 was amplified from PAMs and then inserted into the plasmids pCMV-HA (Clontech, Palo Alto, CA, USA), pCMV-Myc (Clontech) and pWPXL to generate pCMV-HA-ILF2, pCMV-Myc-ILF2 and pWPXL-ILF2, respectively. The nsp2 gene of PRRSV JXwn06 amplified by PCR from the plasmid pWSK-JXwn06 [60] was cloned into the vector pCMV-HA to construct the recombinant plasmid pCMV-HA-nsp2. Three fragments of nsp2 gene were respectively inserted into pWPXL to generate pWPXL-nsp2N (aa1-405), pWPXL-nsp2M (aa323-814) and pWPXL-nsp2C (aa323-1166). The primers used for plasmid construction were listed in Table 1. All recombinant plasmids were further verified by DNA sequencing.

**Table 1:** Primers used in this study

Primers^a^	Sequence (5^′^-3′)^b^	Use
1F	AGCTTTGTTTAAACCCATGTACCCATACGATGTTC	pWPXL-nsp9 construction
1R	CGACGCGTAACTCATGATTGGACCTGAG	
2F	ATGAGGGGGGACAGAGGCCGTG	ILF2 gene amplification
2R	TCACTCCTGAGTCTCCATG	
3F	CCGGAATTCGGATGAGGGGGGACAGAG	pCMV-HA-ILF2/pCMV-Myc-ILF2 construction
3R	CGGGGTACCTCACTCCTGAGTCTCCATG	
4F	AGCTTTGTTTAAACACCATGAGGGGGGACAGAG	pWPXL-ILF2 construction
4R	CGACGCGTAACTCCTGAGTCTCCATG	
5F	CGCGTCGACGGCCGGAAAGAGAGCAAGGA	pCMV-HA-nsp2 construction
5R	GAAGATCTTCATCCCCCTGAAGGCTTCGAA	
6F	AGCTTTGTTTAAACACCATGGCCGGAAAGAGAGCAAGG	pWPXL-nsp2N construction
6R	CGCGACGCGTAATGAAGTCGCCTGGGTGTTGGCTAG	
7F	AGCTTTGTTTAAACACCATGGGCAAGGACTCGGTCCCTCTG	pWPXL-nsp2M construction
7R	CGCGACGCGTAATTGGTCTAAGAGCCTTCCTGC	
8F	AGCTTTGTTTAAACACCATGGGCAAGGACTCGGTCCCTCTG	pWPXL-nsp2C construction
8R	CGCGACGCGTAATCCCCCTGAAGGCTTCGAAATTTGC	

^a^F denotes forward PCR primer; R denotes reverse PCR primer

^b^Restriction sites are underlined

### Lentivirus transduction

The lentivirus packaging system including three plasmids—pWPXL, pMD2.G and psPAX2 (Addgene) was employed. The amplified cDNA of nsp9 or ILF2 gene was cloned into pWPXL and expressed as a GFP-tagged protein. The recombinant lentiviruses that were expressing target protein were rescued according to the method previously described [61]. Briefly, 293FT cells were co-transfected with the three plasmids by using FuGENE HD Transfection Reagent (Promega, Madison, WI, USA) in Opti-MEM (Fisher Scientific). The supernatants were harvested when the cytopathic effect appeared, and were then filtered, concentrated and titrated. 3D4/21 cells or MARC-145 cells were transduced with the recombinant lentiviruses in the presence of polybrene (Sigma).

### IP and Co-IP assays

To explore cellular proteins interacting with the nsp9 of PRRSV and further confirm the interactions, IP and Co-IP assays were performed, respectively. For IP assay, 3D4/21 cells were transduced with the recombinant lentiviruses that were expressing GFP-nsp9 or GFP. At 48 h post-transduction, the cells were harvested in IP buffer containing protease inhibitor cocktail (Sigma) and the supernatants were then collected by centrifugation at 12,000×g for 15 min. After being precleared with protein A/G Sepharose beads (GE Healthcare Bio-Science) for 2 h at 4 °C, the supernatants were precipitated with an anti-GFP mAb in conjunction with protein A/G Sepharose beads. The beads were then washed five times with IP buffer and subjected to boil for 5 min. The proteins isolated from the beads were separated by SDS-PAGE, followed by silver staining or western blotting. For the Co-IP assay of the exogenous ILF2 and nsp9 or nsp2, 293FT cells were transfected with ILF2-, nsp9- or nsp2-expressing plasmid individually or together. At 36 h post-transfection, the cells were harvested, centrifuged, precleared and precipitated with an anti-HA or anti-Myc mAb in conjunction with protein A/G Sepharose beads, followed by SDS-PAGE and western blotting. For the Co-IP assay of the endogenous ILF2 and nsp9 or nsp2, MARC-145 cells infected with the recombinant lentiviruses that were expressing nsp9 or with PRRSV JXwn06 were lysed at 48 h post-transduction or post-infection and then subjected to IP assay as mentioned above.

### Silver staining

The proteins bound to the beads were separated by SDS-PAGE and then visualized using a Pierce™ Silver Stain kit (Fisher Scientific) according to the manufacturer’s instructions. The gels were washed with ultrapure water and fixed in ultrapure water containing 30% ethanol and 10% acetic acid. After being sensitized in Sensitizer Working Solution, the gels were stained with Stain Working Solution. Then, the protein bands were developed with Developer Working Solution and the reaction was stopped with 5% acetic acid in ultrapure water. All differential bands were manually excised from the stained gels and subjected to LC-MS/MS as previously described [59].

### Western blotting

Protein samples were separated by SDS-PAGE, and were electrically transferred onto a polyvinylidenefluoride (PVDF) membrane. After being blocked with 5% skim milk in phosphate-buffered saline (PBS), the membrane was incubated with proper mAb or polyclonal antibody and subsequently probed with appropriate horseradish peroxidase (HRP)-conjugated goat anti-mouse or goat anti-rabbit secondary antibody. The protein bands were developed with the ECL western blotting system (Fisher Scientific) and exposed to a FluorChem E apparatus (ProteinSimple, Santa Clara, CA, USA).

### Confocal immunofluorescence assay

MARC-145 cells and PAMs grown on coverslips in 24-well plates (Costar, Corning Incorporation) were infected with PRRSV JXwn06 at a multiplicity of infection (MOI) of 0.01. Uninfected cells served as mock control. At 36 h post-infection, the cells were fixed and permeabilized with cold anhydrous ethanol for 20 min at room temperature (RT), followed by being blocked with 2% BSA in PBS for 1 h at RT. Then, the cells were incubated with a rabbit anti-ILF2 polyclonal antibody and a mouse anti-nsp9 or anti-nsp2 mAb overnight at 4 °C in a humid chamber. After being rinsed 3 times with PBS, the cells were incubated with fluorescein isothiocyanate (FITC)-conjugated goat anti-rabbit and tetramethyl rhodamine isothiocyanate (TRITC)-conjugated goat anti-mouse secondary antibody for 1 h at RT. Finally, the cells were stained with DAPI, and the images were viewed under an Olympus confocal microscope (Fluoview1000).

### ILF2 gene silencing by small interfering RNA (siRNA)

SiRNAs targeting ILF2 gene were designed and synthesized by the GenePharma (Suzhou, China), and were used to analyze the effect of ILF2 knockdown on the replication of PRRSV. MARC-145 cells grown on six-well plates at 30 ~ 40% confluence were transfected with the siRNA using Lipofectamine RNAiMAX reagent (Fisher Scientific) according to the manufacturer’s protocol. After 48 h post-transfection, the cells were harvested for analysis of silencing efficiency or infected with PRRSV JXwn06.

### The over-expression of ILF2 in MARC-145 cells

To analyze the influence of the ILF2 over-expression on the replication of PRRSV, MARC-145 cells that were expressing ILF2 were established by lentivirus packaging system according to the manufacturer’s protocol. In brief, MARC-145 cells were transduced with the recombinant lentiviruses that were expressing GFP-ILF2 or GFP with 8 μg/ml of polybrene (Sigma). At 48 h post-transduction, the cells were harvested for analysis of ILF2 expression by western blotting or infected with PRRSV JXwn06 for virus titration.

### Viral infection and titration

MARC-145 cells were infected with PRRSV JXwn06 at a MOI of 0.01. Viral titers were titrated by a microtitration infectivity assay as previously described [60].

### Statistical analysis

Data are expressed as means ± standard deviations (SD). The statistically significant differences among groups were evaluated by two-way ANOVA using GraphPad Prism (version 5.0) software. Differences were considered statistically significant at a value of *p* < 0.05.

## Results

### Cellular ILF2 interacted with the nsp9 of PRRSV

**Fig. 1:**
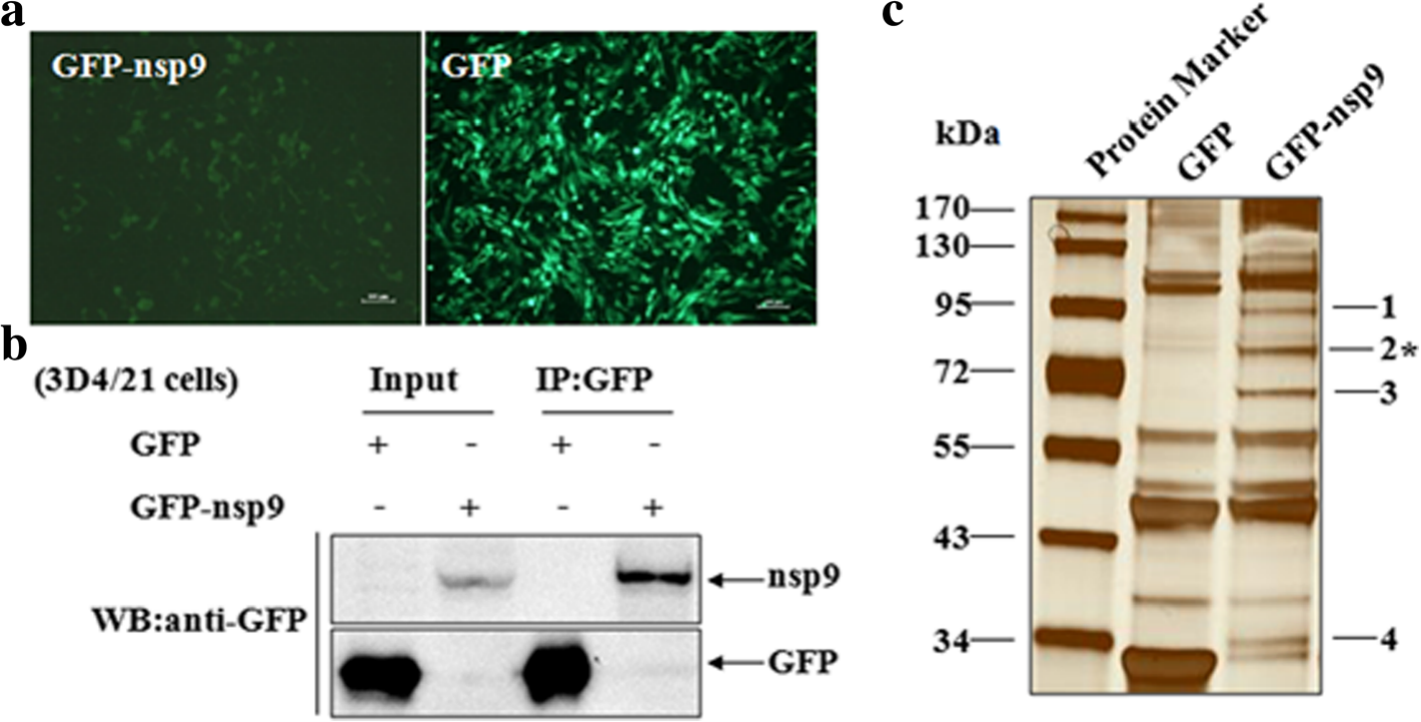
Analysis of cellular proteins interacting with PRRSV nsp9 by IP combined with LC-MS/MS assay. 3D4/21 cells were transduced with GFP-nsp9- or GFP-expressing lentiviruses (**a**) and then the expression of GFP-nsp9 and GFP in 3D4/21 cells were examined by western blotting (**b**). The cell lysates were immunoprecipitated with anti-GFP mAb, and were then separated by SDS-PAGE, and followed by silver staining (**c**). Shown are the differential protein bands between GFP-nsp9- and GFP-expressing 3D4/21 cells. Asterisk indicatess the band from which ILF2 was identified

To identify host cellular proteins interacting with the nsp9 of PRRSV, 3D4/21 cells were transduced with the GFP-nsp9-expressing lentiviruses, and 3D4/21 cells transduced with the GFP-expressing lentiviruses served as a control (Fig. 1a). At 48 h post-infection, the cells were harvested and immunoprecipitated with an anti-GFP mAb. The immunoprecipitated proteins were then subjected to SDS-PAGE, and GFP-nsp9 and GFP were examined with an anti-GFP mAb (Fig. 1b) or visualized using silver staining (Fig. 1c). Compared with the control lane, four bands could be detected in the cells that were over-expressing GFP-nsp9. A total of 52 proteins interacting with the nsp9 of PRRSV were successfully identified by LC-MS/MS from the bands in the cells that were expressing nsp9 of PRRSV (Additional file 1). Of these proteins, ILF2 was chosen for further analysis due to its higher score.

**Fig. 2:**
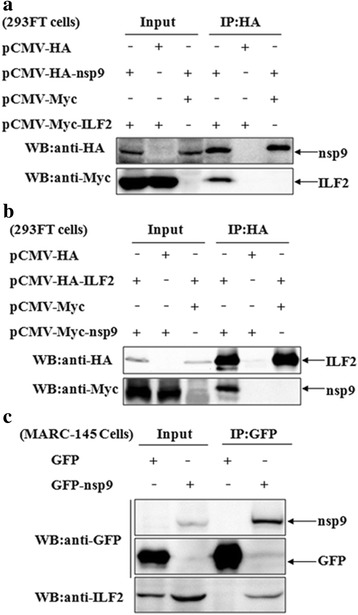
Interaction of the nsp9 with ILF2. **a** and **b** The interaction of nsp9 with exogenous ILF2. 293FT cells were co-transfected with the indicated plasmids. The cell lysates were immunoprecipitated with an anti-HA mAb and detected with an anti-HA mAb or anti-Myc polyclonal antibody. **c** The interaction of nsp9 with endogenous ILF2. MARC-145 cells were transduced with GFP-nsp9- or GFP-expressing lentiviruses. The cell lysates were immunoprecipitated with an anti-GFP mAb and detected with an anti-GFP or anti-ILF2 mAb

To confirm the interaction between ILF2 and nsp9, 293FT cells were transfected with ILF2- and nsp9-expressing plasmid. The cell lysates were immunoprecipitated with an anti-HA mAb and were then subjected to western blotting. It was shown that nsp9 could be pulled down in the cells that were co-transfected with ILF2- and nsp9-expressing plasmid (pCMV-HA-ILF2 and pCMV-Myc-nsp9), whereas it could not be done in the cells that were co-transfected with the plasmids pCMV-HA and pCMV-Myc-nsp9 (Fig. 2a). The reciprocal IP assay was also performed and the same results were observed (Fig. 2b).

To further verify the interaction of endogenous ILF2 with the nsp9 of PRRSV in MARC-145 cells, MARC-145 cells were transduced with GFP-nsp9-expressing lentiviruses. The cells transduced with GFP-expressing lentiviruses served as a control. As shown in Fig. 2c, ILF2 could be only detected in the immunoprecipitated samples of cells that were expressing GFP-nsp9, suggesting that endogenous ILF2 in MARC-145 cells could interact with the nsp9 of PRRSV.

### Cellular ILF2 interacted with the nsp2 of PRRSV

**Fig. 3:**
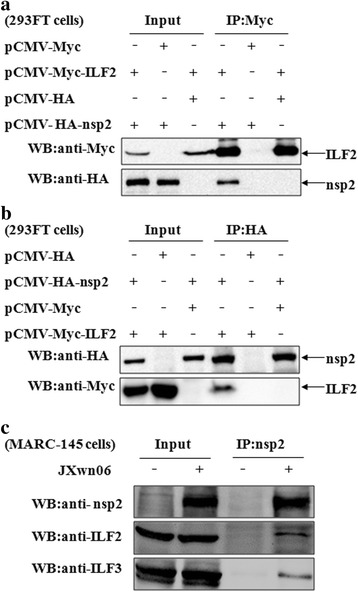
Interaction of the nsp2 with ILF2. **a** and **b** The interaction of nsp2 with exogenous ILF2. 293FT cells were co-transfected with the indicated plasmids. The cell lysates were immunoprecipitated with an anti-HA mAb and further probed with an anti-HA mAb or anti-Myc polyclonal antibody. **c** The interaction of nsp2 with endogenous ILF2 and ILF3. MARC-145 cells were infected with PRRSV JXwn06 and then cell lysates were immunoprecipitated with an anti-nsp2 mAb, and were then detected with an anti-nsp2 or anti-ILF2 mAb or anti-ILF3 polyclonal antibody

ILF2 usually forms a heterodimeric complex with ILF3 [42]. Our previous analysis has indicated the interaction of ILF3 with the nsp2 of PRRSV [58]. Therefore, we speculated that ILF2 might also interact with the nsp2 of PRRSV. To confirm this, 293FT cells were transfected with ILF2- and nsp2-expressing plasmids individually or together. It was shown that ILF2 could be precipitated exclusively from the cells co-transfected with the plasmids pCMV-HA-nsp2 and pCMV-Myc-ILF2 (Fig. 3a). The same results were achieved for the interaction of ILF2 and nsp2 by a reciprocal IP assay (Fig. 3b). Giving this finding, we further examined the interaction of endogenous ILF2 with the nsp2 in the context of PRRSV infection. As shown in Fig. 3c, by Co-IP assay with an anti-nsp2 mAb, ILF2 could only be immunoprecipitated in PRRSV JXwn06-infected MARC-145 cells. In addition, ILF3 could also be pulled down by an anti-nsp2 mAb (Fig. 3c). As a whole, the above results indicated that ILF2 could interact with the nsp2 of PRRSV in MARC-145 cells.

### The regions responsible for the interaction of ILF2 with the nsp9 or nsp2 of PRRSV

**Fig. 4:**
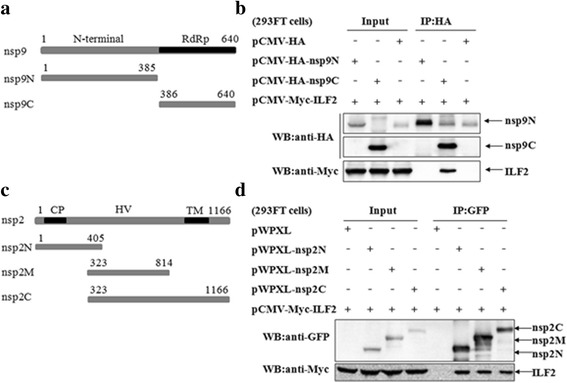
The regions responsible for the interaction of ILF2 with nsp9 and nsp2. **a** The schematic diagram of the truncated nsp9—nsp9N (aa1-385) and nsp9C (aa386-640). **b** The interaction of ILF2 with nsp9C by Co-IP. 293FT cells were co-transfected with the indicated plasmids. The cell lysates were immunoprecipitated with an anti-HA mAb and further probed with an anti-HA or anti-Myc MAb. **c** The schematic diagram of three truncated nsp2—nsp2N (aa1-405), nsp2M (aa323-814) and nsp2C (aa323-1166). **d** The interaction of ILF2 with nsp2N, nsp2M and nsp2C by Co-IP. 293FT cells were co-transfected with the indicated plasmids. The cell lysates were immunoprecipitated with an anti-GFP mAb and further probed with an anti-GFP mAb or anti-Myc polyclonal antibody

The nsp9 of PRRSV has two domains, a C-terminal RdRp domain and N-terminal domain with unknown function [16]. The nsp2 of PRRSV contains a putative cysteine protease (PL2) domain possessing cleavage activity as well as deubiquitinating activity [62–66], a middle hypervariable region with unspecific function and C-terminal transmembrane (TM) domain [67, 68]. To determine the regions of nsp9 and nsp2 responsible for the interaction with ILF2, a serial of truncated mutants, as indicated, expressed as HA- or GFP-fused protein (Fig. 4a and c) were co-transfected with pCMV-Myc-ILF2 in 293FT cells. The cell lysates were precipitated with an anti-HA or anti-GFP mAb. The results showed that the truncated nsp9C retained the ability to interact with ILF2, while the truncated nsp9N did not (Fig. 4b), indicating that the RdRp domain of nsp9 is responsible for its interaction with ILF2. However, all three truncated nsp2 (nsp2N, nsp2M and nsp2C) were able to interact with ILF2 (Fig. 4d).

### ILF2 partly co-localized with the nsp9 or nsp2 of PRRSV in the cytoplasm

**Fig. 5:**
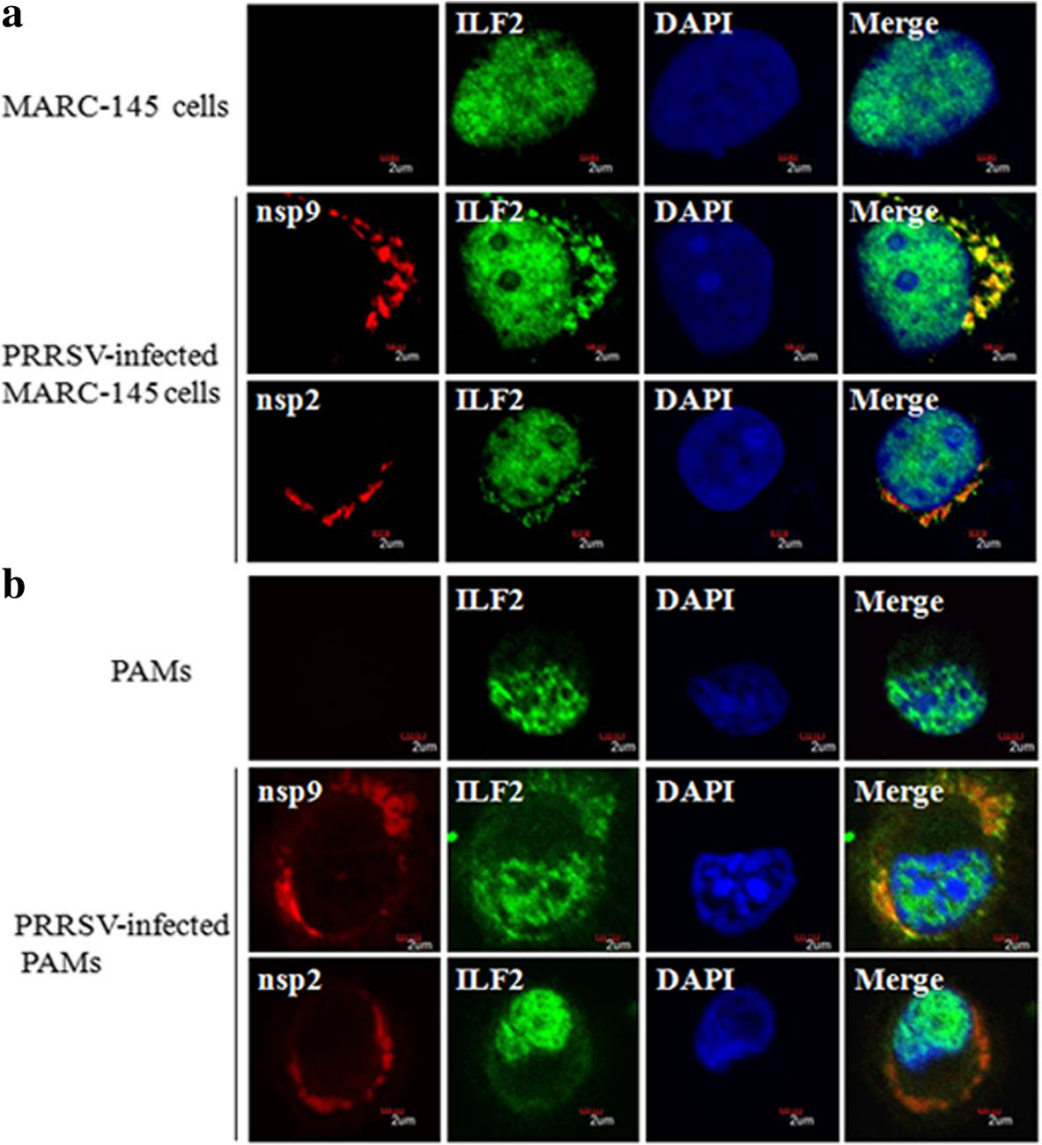
Co-localization of ILF2 with nsp9 and nsp2 in MARC-145 cells (**a**) and PAMs (**b**). The cells were infected with PRRSV JXwn06 at a MOI of 0.01, and were then fixed and double-stained at 36 h post-infection with a rabbit anti-ILF2 polyclonal antibody and a mouse anti-nsp9 or anti-nsp2 mAb, and followed by the FITC-conjugated goat anti-rabbit IgG (*green*) and TRITC-conjugated goat anti-mouse IgG (*red*). Nuclei were stained with DAPI

Having demonstrated that ILF2 was able to interact with the nsp9 or nsp2 of PRRSV by Co-IP, we further analyzed whether ILF2 co-localizes with nsp9 or nsp2 in PRRSV-infected cells. MARC-145 cells were infected with PRRSV JXwno6 at a MOI of 0.01, and confocal immunofluorescence assay was performed. As shown in Fig. 5a, ILF2 partly could be translocated from the nucleus to the cytoplasm and co-localized in the cytoplasm with the nsp9 or nsp2 in PRRSV-infected MARC-145 cells, while ILF2 extremely localized in the nucleus of mock-infected MARC-145 cells. Similar phenomenon could be observed in PRRSV-infected PAMs (Fig. 5b).

### Knockdown of ILF2 favored the replication of PRRSV in MARC-145 cells

**Fig. 6:**
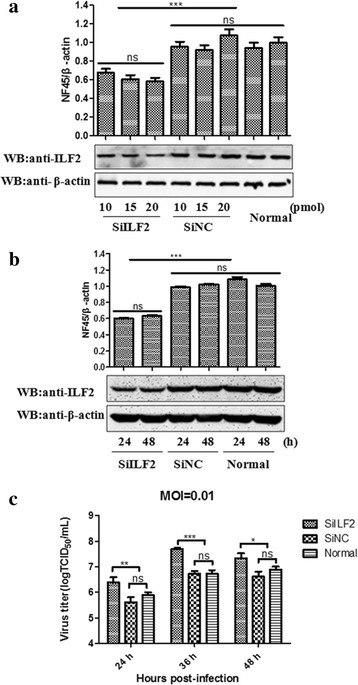
Enhancement of PRRSV replication by ILF2 knockdown in MARC-145 cells. **a** MARC-145 cells were transfected with different concentrations of siRNAs targeting ILF2 (SiILF2) and the silencing efficiency of ILF2 was examined by western blotting with an anti-ILF2 mAb. The quantity of β-actin was used for normalization of the amount of ILF2 expression. The optical density ratios of ILF2/β-actin are shown with graphs. MARC-145 cells transfected with SiILF2 or negative control siRNA (SiNC), and normal MARC-145 cells, were infected with PRRSV JXwn06 at a MOI of 0.01. The silencing efficiency (**b**) and the virus titers were examined (**c**) at the indicated time points post-infection. Data are expressed as means ± SD of three independent experiments (*p < 0.05; ** *p* < 0.01; *** *p* < 0.001; ns, no significant)

The effect of ILF2 knockdown on the replication of PRRSV was analyzed by using siRNAs. MARC-145 cells were transfected with the siRNAs targeting ILF2 and were then harvested for examining the expression of ILF2 or were infected with PRRSV JXwn06 for virus titration. Compared with the normal cells or cells transfected with control siRNAs (SiNC), the cells transfected with ILF2-specific siRNAs (SiILF2) at a final concentration of 20 pmol exhibited a significantly decreased level of ILF2 expression (Fig. 6a and b) (*p* < 0.001). There was a 5.99-fold, 9.52-fold and 5.27-fold increase of the virus yields in the SiILF2-transfected cells at 24 h (*p* < 0.01), 36 h (*p* < 0.001), 48 h (*p* < 0.05) post-infection, respectively (Fig. 6c), suggesting that the knockdown of ILF2 favors the replication of PRRSV in MARC-145 cells.

### Over-expression of ILF2 impacted the replication of PRRSV in MARC-145 cells

**Fig. 7:**
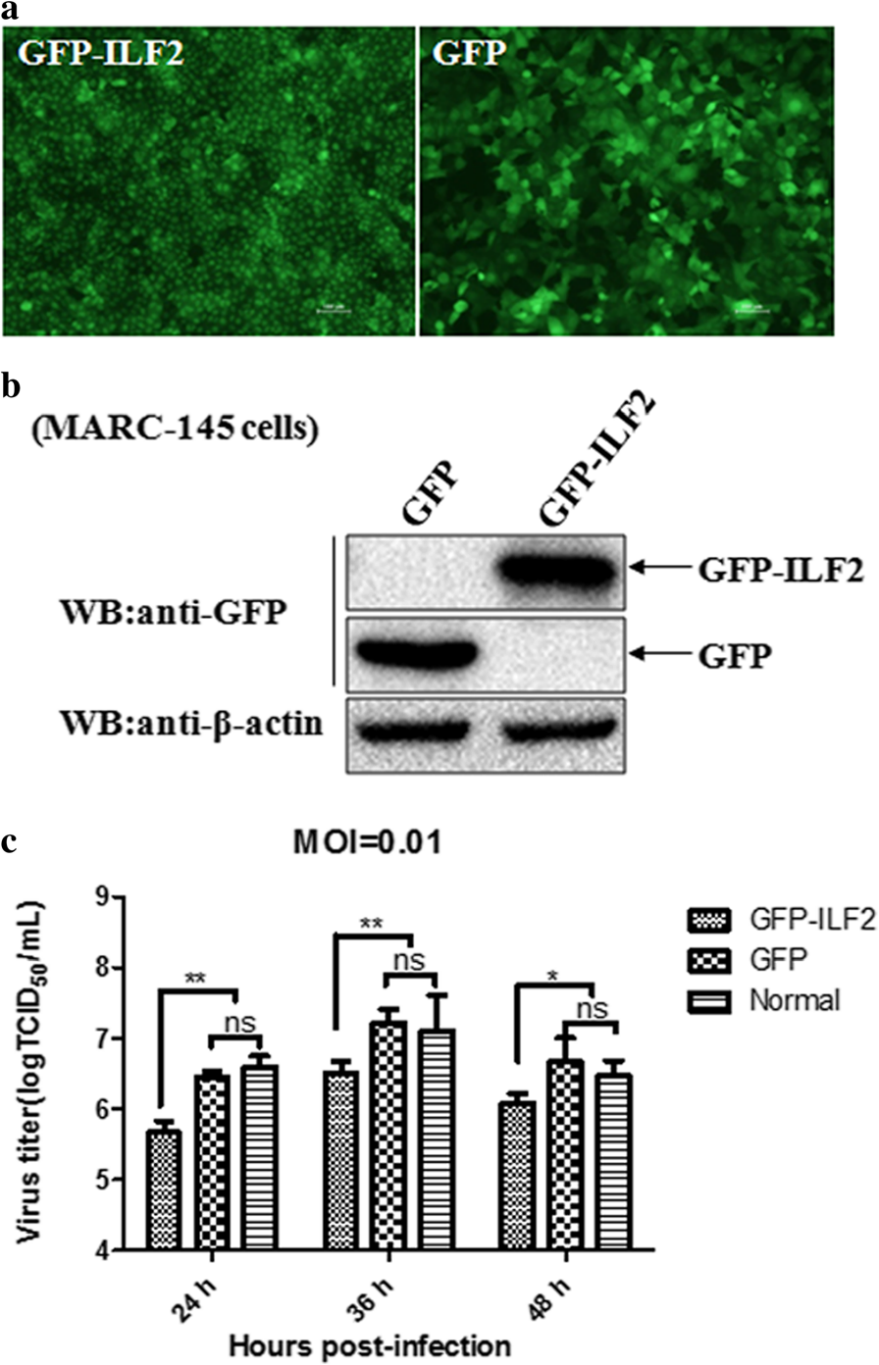
Inhibition of PRRSV replication by ILF2 over-expression in lentiviruses- transduced MARC-145 cells. Lentiviruses that were expressing GFP-ILF2 or GFP were transduced into MARC-145 cells (**a**) and then the expression of GFP-nsp9 and GFP in MARC-145 cells were examined by western blotting (**b**). The β-actin was used for normalization of the expression of ILF2. The transduced MARC-145 cells were infected with PRRSV JXwn06 at a MOI of 0.01. Normal MARC-145 cells were infected with PRRSV JXwn06 as a control. The virus yields were assayed at the indicated time points post-infection (**c**). Data are expressed as means ± SD of three independent experiments (*p < 0.05; ** *p* < 0.01; ns, no significant)

The effect of ILF2 over-expression on the replication of PRRSV was examined in MARC-145 cells. MARC-145 cells were transduced with the GFP-ILF2- or GFP- expressing lentiviruses, and followed by western blotting for analysis of ILF2 expression or infected with PRRSV JXwn06 for virus titration. The results showed that the expression of GFP-ILF2 could be observed (Fig. 7a) and the expression level of GFP-ILF2 was similar to GFP (Fig. 7b). Over-expression of ILF2 resulted in a significantly decrease of virus titers in PRRSV-infected MARC-145 cells at 24 h (*p* < 0.01), 36 h (*p* < 0.01), 48 h (*p* < 0.05) post-infection (Fig. 7c), suggesting that the over-expression of ILF2 impacts the replication efficiency of PRRSV.

## Discussion

Viruses can rely on host cellular proteins to complete its infection cycle, and reversely some cellular proteins play important roles in restricting viral replication. The interactions between cellular proteins and viral proteins are involved in the regulation of viral replication. The nsp9, as the RdRp of PRRSV, has been considered to play essential roles in the replication efficiency in vitro and in vivo and the fatal virulence for piglets of the Chinese highly pathogenic PRRSV [33]. Previous studies have demonstrated that several cellular proteins could interact with the nsp9 of PRRSV and their interactions conferred to the regulation of viral replication [34–36]. The nsp2 of PRRSV is generally recognized as a multifunctional protein in viral replication and pathogenesis [37, 38]. In the present study, we found porcine cellular ILF2 interacting with the nsp9 of PRRSV by Co-IP combined with LC-MS/MS assay. Then, we employed Co-IP assay to further confirm this interaction in both 293FT cells and MARC-145 cells. Meanwhile, we verified the interaction of ILF2 with the nsp2 of PRRSV. Thus, ILF2 could interact with two nsps of PRRSV—nsp9 and nsp2, which are considered as parts of the viral RTC [16]. The nsp9 is responsible for RNA synthesis as the RdRp, while nsp2, together with nsp3, form the DMVs, which provide the sites for viral RNA synthesis [39, 69]. A similar scenario has been described by Stricker et al. in the case of IBDV [50], showing ILF2 can interact with the viral RdRp VP1, the capsid protein VP2, and the ribonucleoprotein VP3 in different cell lines. Thus, we proposed that the cellular protein ILF2 possibly participates in the RTC of PRRSV either by the viral exploitation or as a host defense mechanism. Certainly, further investigation is required to support this hypothesis.

The cellular proteins usually localize in appropriate subcellular compartments to execute their biological functions. Once the cells are infected with viruses, some cellular proteins can re-locate in favor of viral replication or against viral replication. In IBDV-infected cells, ILF2 gradually has been shown to be translocated from the nucleus to the cytoplasm, and cytoplasmic ILF2 accumulates at the sites of viral replication where VP1, VP2, and VP3 localize [50]. Similar findings are described in HCV, indicating that ILF2 not only co-localizes with viral NS5A, but also interacts with the viral genome RNA [70]. Similarly, our findings revealed that the cellular ILF2 could be translocated partly from the nucleus to the cytoplasm and co-localized with the nsp9 and nsp2 of PRRSV in PRRSV-infected MARC-145 cells and PAMs, while ILF2 only localized in the nucleus in non-infected cells. Thus, we proposed that during PRRSV infection the translocation of ILF2 from the nucleus to the cytoplasm is likely involved in the formation of viral replication complexes (RTC), and successively exerts the biological effect of regulating the replication of PRRSV. In spite of the fact that ILF2 interacts with the genomic RNA of HCV [70], whether ILF2 interacts with genomic RNA and/or subgenomic RNA of PRRSV is required to be further explored.

To address the biological significance of interaction between ILF2 with the nsp9 and nsp2 on the replication of PRRSV, we analyzed the effect of ILF2 knockdown by siRNA silencing and ILF2 over-expression by the lentivirus packaging system on the replication of PRRSV in MARC-145 cells. Our results indicated that ILF2 knockdown favored the viral replication, while ILF2 over-expression impaired the viral replication, suggesting that ILF2 play negatively regulatory effect on the replication of PRRSV. Previous studies have shown that ILF2 silencing promotes the replication of IBDV [50], and knockdown of ILF3—the ILF2 partner also enhances the replication of vesicular stomatitis virus [57] and influenza virus [52]. It is proposed that ILF2 might act as a restriction factor for PRRSV replication by exerting regulatory effect on the genome transcription or protein expression of PRRSV, but the exact mechanism concerning the role of ILF2 in the replication process of PRRSV needs to be done in the future.

## Conclusion

As a whole, our findings are the first to confirm that the porcine ILF2 interacts with the nsp9 and nsp2 of PRRSV in vitro, and exerts negatively regulatory effect on the replication of PRRSV. Our present study provides more evidence for understanding the roles of the interactions between cellular proteins and viral proteins in the replication of PRRSV.

## Additional file

Additional file 1: The list of cellular proteins interacting with PRRSV nsp9. (DOCX 22 kb)

## Abbreviations

ILF2: Interleukin-2 enhancer binding factor 2
nsp2: nonstructural protein 2
nsp9: nonstructural protein 9
PAMs: Pulmonary alveolar macrophages
PRRSV: Porcine reproductive and respiratory syndrome virus
