# Supplementary material for: Interleukin-2 enhancer binding factor 2 interacts with the nsp9 or nsp2 of porcine reproductive and respiratory syndrome virus and exerts negatively regulatory effect on the viral replication
Source: Virol J. 2017 Jul 11;14:125. doi: 10.1186/s12985-017-0794-5 (PMC5504599; doi:10.1186/s12985-017-0794-5)
Supplement: Additional file 1: — The list of cellular proteins interacting with PRRSV nsp9. (DOCX 22 kb) [file 12985_2017_794_MOESM1_ESM.docx]

**Table S1. The list of cellular proteins interacting with PRRSV Nsp9**

| ID | Score | MW (D) | Protein name |
| --- | --- | --- | --- |
| Protein band 1 |  |  |  |
| [gi\|999627](http://localhost/mascot/cgi/master_results.pl?file=../data/20130810/F015234.dat) | 36 | 8814 | Chain B, Refined 1.8 Angstroms Resolution Crystal Structure Of Porcine Epsilon-Trypsin |
| [gi\|335294269](http://localhost/mascot/cgi/master_results.pl?file=../data/20130810/F015234.dat) | 31 | 51022 | PREDICTED: ribosomal RNA-processing protein 8 [Sus scrofa] |
| [gi\|238065895](http://localhost/mascot/cgi/master_results.pl?file=../data/20130810/F015234.dat) | 30 | 43542 | RecName: Full=Actin-binding Rho-activating protein; AltName: Full=Striated muscle activator of Rho--dependent signaling; Short=STARS |
| [gi\|209863051](http://localhost/mascot/cgi/master_results.pl?file=../data/20130810/F015234.dat) | 30 | 43542 | actin-binding Rho-activating protein [Sus scrofa] |
| [gi\|108735896](http://localhost/mascot/cgi/master_results.pl?file=../data/20130810/F015234.dat) | 30 | 43542 | striated muscle activator of Rho-dependent signaling [Sus scrofa] |
| [gi\|335286080](http://localhost/mascot/cgi/master_results.pl?file=../data/20130810/F015234.dat) | 29 | 122615 | PREDICTED: DNA topoisomerase I, mitochondrial-like [Sus scrofa] |
| [gi\|62510704](http://localhost/mascot/cgi/master_results.pl?file=../data/20130810/F015234.dat) | 29 | 44866 | RecName: Full=Phosphoglycerate kinase 2; AltName: Full=Phosphoglycerate kinase, testis specific |
| [gi\|47523276](http://localhost/mascot/cgi/master_results.pl?file=../data/20130810/F015234.dat) | 29 | 44866 | phosphoglycerate kinase 2 [Sus scrofa] |
| [gi\|40748088](http://localhost/mascot/cgi/master_results.pl?file=../data/20130810/F015234.dat) | 29 | 44817 | testis-specific phosphoglycerate kinase [Sus scrofa] |
| [gi\|51247812](http://localhost/mascot/cgi/master_results.pl?file=../data/20130810/F015234.dat) | 29 | 13284 | Chain A, Crystal Structure Of Porcine Alpha Trypsin Bound With Auto Catalyticaly Produced Native Peptide At 2.15 A Resolution |
| [gi\|1942351](http://localhost/mascot/cgi/master_results.pl?file=../data/20130810/F015234.dat) | 29 | 13284 | Chain A, Crystal Structure Of The First Active Autolysate Form Of The Porcine Alpha Trypsin |
| [gi\|335306566](http://localhost/mascot/cgi/master_results.pl?file=../data/20130810/F015234.dat) | 29 | 14691 | PREDICTED: membrane magnesium transporter 1-like [Sus scrofa] |
| [gi\|311272045](http://localhost/mascot/cgi/master_results.pl?file=../data/20130810/F015234.dat) | 28 | 37242 | PREDICTED: glutaredoxin-3-like [Sus scrofa] |
| [gi\|338784423](http://localhost/mascot/cgi/master_results.pl?file=../data/20130810/F015234.dat) | 28 | 175816 | eukaryotic translation initiation factor 4 gamma 1 [Sus scrofa] |
| Protein band 2 |  |  |  |
| [gi\|311254260](http://localhost/mascot/cgi/master_results.pl?file=../data/20130810/F015236.dat) | 99 | 43023 | PREDICTED: interleukin enhancer-binding factor 2 [Sus scrofa] |
| [gi\|311265678](http://localhost/mascot/cgi/master_results.pl?file=../data/20130810/F015236.dat) | 47 | 42059 | PREDICTED: alpha-2,8-sialyltransferase 8F-like [Sus scrofa] |
| [gi\|311268697](http://localhost/mascot/cgi/master_results.pl?file=../data/20130810/F015237.dat) | 57 | 144481 | PREDICTED: LOW QUALITY PROTEIN: tetratricopeptide repeat protein 21A-like [Sus scrofa] |
| [gi\|335285840](http://localhost/mascot/cgi/master_results.pl?file=../data/20130810/F015237.dat) | 52 | 299963 | PREDICTED: apolipoprotein B-100-like [Sus scrofa] |
| [gi\|5052383](http://localhost/mascot/cgi/master_results.pl?file=../data/20130810/F015238.dat) | 42 | 19939 | glyceraldehyde-3-phosphate dehydrogenase GAPDH [Sus scrofa] |
| [gi\|311265357](http://localhost/mascot/cgi/master_results.pl?file=../data/20130810/F015238.dat) | 34 | 19052 | PREDICTED: EF-hand calcium-binding domain-containing protein 2-like [Sus scrofa] |
| [gi\|335281134](http://localhost/mascot/cgi/master_results.pl?file=../data/20130810/F015238.dat) | 34 | 122155 | PREDICTED: rap guanine nucleotide exchange factor 1 [Sus scrofa] |
| [gi\|335282347](http://localhost/mascot/cgi/master_results.pl?file=../data/20130810/F015238.dat) | 32 | 67914 | PREDICTED: lamin-B2 [Sus scrofa] |
| [gi\|38098884](http://localhost/mascot/cgi/master_results.pl?file=../data/20130810/F015238.dat) | 28 | 10340 | testis-specific phosphoglycerate kinase 2 [Sus scrofa] |
| [gi\|311274155](http://localhost/mascot/cgi/master_results.pl?file=../data/20130810/F015238.dat) | 28 | 13321 | PREDICTED: NADH dehydrogenase [ubiquinone] iron-sulfur protein 6, mitochondrial-like [Sus scrofa] |
| [gi\|149132072](http://localhost/mascot/cgi/master_results.pl?file=../data/20130810/F015240.dat) | 66 | 16387 | multiprotein bridging factor 1 [Sus scrofa] |
| [gi\|47523888](http://localhost/mascot/cgi/master_results.pl?file=../data/20130810/F015240.dat) | 47 | 32889 | ADP/ATP translocase 3 [Sus scrofa] |
| [gi\|42565038](http://localhost/mascot/cgi/master_results.pl?file=../data/20130810/F015240.dat) | 47 | 32889 | mitochondrial solute carrier family 25 member 6 [Sus scrofa] |
| [gi\|335298625](http://localhost/mascot/cgi/master_results.pl?file=../data/20130810/F015240.dat) | 37 | 37733 | PREDICTED: leucine-rich repeat-containing protein C17orf76-like [Sus scrofa] |
| [gi\|311262501](http://localhost/mascot/cgi/master_results.pl?file=../data/20130810/F015240.dat) | 35 | 46929 | PREDICTED: methylmalonic aciduria type A protein, mitochondrial [Sus scrofa] |
| [gi\|31075434](http://localhost/mascot/cgi/master_results.pl?file=../data/20130810/F015240.dat) | 33 | 12545 | adipocyte determination and differentiation-dependent factor 1 [Sus scrofa] |
| [gi\|155604159](http://localhost/mascot/cgi/master_results.pl?file=../data/20130810/F015240.dat) | 33 | 10942 | MHC class II antigen [Sus scrofa] |
| [gi\|297747346](http://localhost/mascot/cgi/master_results.pl?file=../data/20130810/F015240.dat) | 33 | 11437 | S100 calcium binding protein A14 [Sus scrofa] |
| [gi\|335293323](http://localhost/mascot/cgi/master_results.pl?file=../data/20130810/F015240.dat) | 33 | 81777 | PREDICTED: stromal interaction molecule 2-like [Sus scrofa] |
| [gi\|335281027](http://localhost/mascot/cgi/master_results.pl?file=../data/20130810/F015240.dat) | 32 | 65982 | PREDICTED: syntaxin-binding protein 1-like [Sus scrofa] |
| Protein band 3 |  |  |  |
| [gi\|335302749](http://localhost/mascot/cgi/master_results.pl?file=../data/20130810/F015242.dat) | 110 | 48392 | PREDICTED: heterogeneous nuclear ribonucleoproteins A2/B1-like [Sus scrofa] |
| [gi\|335295838](http://localhost/mascot/cgi/master_results.pl?file=../data/20130810/F015242.dat) | 49 | 34789 | PREDICTED: major histocompatibility complex class I-related gene protein-like isoform 2 [Sus scrofa] |
| [gi\|335304007](http://localhost/mascot/cgi/master_results.pl?file=../data/20130810/F015242.dat) | 47 | 260782 | PREDICTED: microtubule-associated serine/threonine-protein kinase 4, partial [Sus scrofa] |
| [gi\|311274365](http://localhost/mascot/cgi/master_results.pl?file=../data/20130810/F015242.dat) | 42 | 86847 | PREDICTED: ankyrin repeat domain-containing protein 5 [Sus scrofa] |
| [gi\|335301228](http://localhost/mascot/cgi/master_results.pl?file=../data/20130810/F015242.dat) | 41 | 19631 | PREDICTED: kinase suppressor of Ras 2-like [Sus scrofa] |
| [gi\|311275644](http://localhost/mascot/cgi/master_results.pl?file=../data/20130810/F015242.dat) | 36 | 49420 | PREDICTED: t-box transcription factor TBX20-like [Sus scrofa] |
| Protein band 4 |  |  |  |
| [gi\|335310341](http://localhost/mascot/cgi/master_results.pl?file=../data/20130810/F015233.dat) | 35 | 15483 | PREDICTED: protein FAM176B-like [Sus scrofa] |
| [gi\|335297607](http://localhost/mascot/cgi/master_results.pl?file=../data/20130810/F015233.dat) | 26 | 85147 | PREDICTED: breast cancer type 1 susceptibility protein homolog isoform 2 [Sus scrofa] |
| [gi\|335287636](http://localhost/mascot/cgi/master_results.pl?file=../data/20130810/F015233.dat) | 25 | 23018 | PREDICTED: intraflagellar transport protein 27 homolog [Sus scrofa] |
| [gi\|45269017](http://localhost/mascot/cgi/master_results.pl?file=../data/20130810/F015233.dat) | 24 | 6098 | fuse-binding protein-interacting repressor SIAHBP1 [Sus scrofa] |
| [gi\|311251123](http://localhost/mascot/cgi/master_results.pl?file=../data/20130810/F015233.dat) | 23 | 27750 | PREDICTED: vacuolar protein sorting-associated protein 37D-like [Sus scrofa] |
| [gi\|335282629](http://localhost/mascot/cgi/master_results.pl?file=../data/20130810/F015233.dat) | 23 | 25076 | PREDICTED: queuine tRNA-ribosyltransferase [Sus scrofa] |
| [gi\|21586150](http://localhost/mascot/cgi/master_results.pl?file=../data/20130810/F015233.dat) | 23 | 3764 | TCR-delta chain CDR3 region [Sus scrofa] |
| [gi\|335283593](http://localhost/mascot/cgi/master_results.pl?file=../data/20130810/F015233.dat) | 23 | 57267 | PREDICTED: histidyl-tRNA synthetase, cytoplasmic-like [Sus scrofa] |
| [gi\|29420628](http://localhost/mascot/cgi/master_results.pl?file=../data/20130810/F015233.dat) | 23 | 18281 | T cell receptor alpha chain [Sus scrofa] |
| [gi\|62526591](http://localhost/mascot/cgi/master_results.pl?file=../data/20130810/F015233.dat) | 23 | 33998 | cyclin G1 [Sus scrofa] |
| [gi\|262204916](http://localhost/mascot/cgi/master_results.pl?file=../data/20130810/F015233.dat) | 22 | 19520 | pyruvate dehydrogenase kinase isozyme 3 [Sus scrofa] |
| [gi\|335284456](http://localhost/mascot/cgi/master_results.pl?file=../data/20130810/F015233.dat) | 22 | 6338 | PREDICTED: leucine carboxyl methyltransferase 1-like [Sus scrofa] |
